# Cancer control in Africa: is cancer research a luxury or necessity?

**DOI:** 10.3332/ecancer.2019.947

**Published:** 2019-07-25

**Authors:** Twalib Ngoma, Mamsau Ngoma

**Affiliations:** 1 Department of Clinical Oncology, Muhimbili University of Health and Allied Sciences (MUHAS), PO box 65001, Dar es Salaam, Tanzania; 2 Academic Unit, Ocean Road Cancer Institute, Dar es Salaam, Tanzania

**Keywords:** cancer, research

## Abstract

Decision makers dealing with resource allocation in Africa have the impression that cancer research is a luxury. As a result of this, very few or no resources are allocated for cancer research in Africa. Since in healthcare, clinical and epidemiological research provides an evidence base for formulation of health policies and facilitates decision making by policy makers, the lack of evidence base makes decision making intuitive. A situation like this is not cost-effective and is unacceptable. It is, therefore, important that for Africa to make effective decisions to improve the health of its population, cancer research informing policy and decision makers is a necessity and not a luxury.

## Introduction

The use of research to provide evidence to support the development of cancer-control plans in Africa would appear, at first sight, not to require any justification [1, 2]. However, the impression that decision makers dealing with resource allocation have is that cancer research is a luxury and not a necessity, hence very few or no resources are allocated for cancer research in Africa.

In the 1990s, the disparity in health resources allocation was highlighted by what is now widely known as the 90/10 gap, representing the fact that only 10% of the world’s expenditures on health research were applied to problems in the developing world, which is home for 80%–90% of the world population [11].

As a consequence of the under-investment in the health concerns of developing countries and the widening gap between developed and developing countries, international foundations were established to promote health research in the 1990s [3].

The Global Forum for Health Research (GFHR) was established as a foundation to support a full spectrum of studies, including biomedical and health systems research as well as ancillary health concerns such as health economics and studies in behavioural/social sciences [4]; the Council on Health Research for Development was established in 1993 to strengthen the research capacity of 52 developing countries [5].

In 2001, developing countries spent US$106 billion on health research but this was still an under-investment in the health concerns of the developing countries. The under-investment was greatest in the African countries [3].

In April 2001, the African Union countries met in Abuja, Nigeria and pledged to set a target of allocating at least 15% of their annual budget to improve the health sector and urged donor countries to scale up support. Despite the 2001 Abuja Declaration, only four countries in the region met that target by 2014. At the same time, some countries, for example, Tanzania, pledged to set aside 1% of their annual budget for research. This target has not yet been realised more than 15 years later. One wonders whether this is because of lack of resources or lack of prioritisation [6].

There is no argument that research and development cannot be separated and that both require financial resources. The estimated health research requirements needed to sustain the public health sector in Africa is conservatively estimated at US$5billion, but only a fraction of this amount is available for research [3]. This explains why institutions in African countries have low research output. Lack of financial resources is not the only reason; there is also a lack of human resources with specialist training in cancer research. This is a critical area which needs to be addressed urgently as Africa embarks on the path to increase its research output. It is encouraging that there are new and emerging opportunities to make progress in this critical area both locally and internationally. At the country level, medical research councils or their equivalents have non-communicable diseases on their agenda for research priorities. There is the African Organisation for Research and Training, which is at the forefront in building cancer research capacity and facilitating cancer research in the continent [7]. The establishment of the New Partnership for Africa’s Development (NEPAD) is a step in the right direction [8]. The central goal of NEPAD is the development of social capital through the training of scientists and support for locally peer-reviewed programmes to improve health services research in Africa.

In addition to the local opportunities, there are many international organisations which, subject to being competitive and meeting the criteria required to qualify for their grant announcements, can award grants to advance cancer research in Africa.

The US National Institutes of Health have many opportunities for cancer research and the US National Cancer Institute Center for Global Health has been supporting Africa to advance global cancer research, build expertise and leverage resources to address the challenges of cancer and reduce cancer deaths for a number of years [9].

The IARC Global Initiative for Cancer Registry Development in Low- and Middle-Income Countries has regional hubs that provide technical and scientific support, deliver tailored training for population based cancer registries, advocate for cancer registration and coordinate international research projects [12] Furthermore, the international Prevention Research Institute in Lyon (iPRI) has a mission to contribute to the improvement of health through research worldwide—this includes Africa [10].

As we can see, there are many organisations which are willing to partner with African institutions to build capacity for cancer research in Africa. What cancer researchers in Africa and their partners need is to see and seize the opportunities available and play their part in the partnerships established. The question as to whether cancer research is a luxury or necessity should be laid to rest and be replaced by the what, why and how questions regarding cancer research in Africa.

## Cancer research in Africa—what is it, why do we need it and how can we do it

Research can be defined in a number of different ways. In common parlance, research refers to a search for knowledge.

In the context of cancer-control planning, research is a systematic way of gathering data and harnessing scientific inquiry to provide scientific information which can be used as the evidence for the recommended objectives, strategies and activities in the cancer plan [2]. We need cancer research because it provides the evidence base on which cancer prevention and control planning strategies are built. Without research data, it is not possible to achieve appropriate targeted preventive or treatment strategies, efficiently use limited healthcare resources, and empower people, healthcare systems and populations to integrate knowledge of disease in improving their health outcomes. In addition to this, development of cancer researchers and research infrastructure stimulates educational, social and economic activities, and thus provides wider benefits to society. With all of these benefits, one would anticipate that collecting data and conducting research to develop informed evidence-based cancer-control policies would rank as one of the highest priorities for all those involved in formulating policy in the public health field. Unfortunately, for most African countries this is not the case—basic statistics such as the burden and geographic distribution of cancer, the prevalence of major risk factors and the health gain introduced by prevention policies are complete unknowns. What happens is that, when creating cancer-control plans, cancer-control workers in these countries have to either operate in an evidence-free black hole or to start to gather data for research and accumulate evidence in the early stages on the pathway of cancer control. The types of cancer research that can be undertaken in Africa are basic, epidemiologic and clinical.

## Basic research

Basic research generates knowledge from the lab/bench which can be useful at the clinical bedside. Curiosity about the unknown is the main motivation for basic research. Usually, the research is performed without thought about practical ends but in the end, it generates knowledge which provides scientific capital and a fund from which practical applications of the knowledge is drawn. In short, basic research is a pacemaker for technological progress.

## Epidemiological research

Epidemiological research generates hypotheses about potential interventions that could reduce cancer incidence or morbidity. Observational epidemiological studies show associations between risk factors and specific cancers while randomised controlled trials test whether hypotheses generated by epidemiological studies and laboratory research actually result in reduced cancer incidence and mortality.

## Clinical research

Clinical research gathers clinical information on diagnosis, treatment and palliation responses. The main motivation is to use the information to determine better health outcomes. The results from this kind of research must be continually evaluated to determine their efficacy, cost-effectiveness and reproducibility.

## Steps in conducting cancer research

The steps in conducting cancer research include:

- Identification of cancer problem
- Literature review
- Specifying the purpose of research
- Determining specific research questions
- Choosing the methodology for data collection such as CANREG4
- Data collection
- Verifying data
- Analysing and interpreting the data
- Reporting and evaluating research
- Communicating the research findings and recommendations

The ultimate goal of these steps is to decrease the incidence of cancer and improve the effectiveness of interventions to screen, detect, treat and palliate cancer.

## The challenges encountered by researchers in Africa

Researchers in Africa face several challenges which have to be addressed to solve the low research output in the continent. These challenges are:

1. *Lack of scientific training in the methodology of research*—this leads to no insight shed on the collated materials.
2. *Insufficient interaction between university research departments and government departments*—this leads to academics not getting ideas from health policy makers on what needs to be researched so that the policy makers can apply the research done by academics.
3. *Lack of confidence on the research findings by the government and other users of research findings*—this leads to research findings not being implemented and/or largely ignored after publication.
4. *Duplication of research studies*—this is a waste of meagre research resources.
5. *Lack of code of conduct for researchers*—this leads to inter-university and interdepartmental rivalries.
6. *Lack of adequate administrative assistance*— this is especially applicable to secretarial, financial and computer assistance.
7. *Lack of library support*—this leads to a lot of time and energy of researchers being spent on tracing out books, journals, reports, etc., rather than in tracing out relevant material from them.
8. *Language barrier*—the languages of communication in research are English or French. These languages are not the mother tongues for Africa. Imagine an Englishman applying for a research grant in Swahili, Arabic, Hausa, Gujarati or Chinese. What do you think would be the odds of being awarded a grant?

## Conclusion

Research and development are inseparable. There is a saying that all progress is born out of inquiry because inquiry leads to research which inculcates scientific and inductive thinking promoting the development of logical habits of thinking and organisation. In healthcare, logical thinking provides the basis for health policies and facilitates decision making by policy makers. They replace intuitive health decisions which are not cost-effective in most cases. Research is therefore a necessity rather than a luxury in Africa because it is needed to inform policy and decision makers so that they make cost-effective decisions to improve the health of their populations.﻿

## Conflicts of interest

The authors have no conflicts of interest.

## Funding declaration

The authors did not receive any funding regarding this article.
